# Correction: Association of daytime napping with incidence of chronic kidney disease and end-stage kidney disease: A prospective observational study

**DOI:** 10.1371/journal.pone.0311422

**Published:** 2024-09-27

**Authors:** Qinjun Li, Ying Shan, Jingchi Liao, Ling Wang, Yanling Wei, Liang Dai, Sen Kan, Jianqing Shi, Xiaoyan Huang, Guoyuan Lu

There are errors in the author affiliations. The correct affiliations are as follows:

Qinjun Li^1,2^, Ying Shan^3^, Jingchi Liao^4^, Ling Wang^2^, Yanling Wei^3^, Liang Dai^3^, Sen Kan^2^, Jianqing Shi^4,5^, Xiaoyan Huang^3,6^, Guoyuan Lu^1^

1 Department of Nephrology, The First Affiliated Hospital of Soochow University, Suzhou, Jiangsu, China, **2** Department of Nephrology, The Affiliated Xuzhou Municipal Hospital of Xuzhou Medical University, Xuzhou, Jiangsu, China, **3** Department of Clinical Research Academy, Peking University Shenzhen Hospital, Shenzhen, Guangdong, China, **4** Department of Statistics and Data Science, Southern University of Science and Technology, Shenzhen, Guangdong, China, **5** Department of Statistics and Data Science, National Center for Applied Mathematics, Shenzhen, Guangdong, China, **6** Renal Division, Peking University Shenzhen Hospital, Shenzhen, Guangdong, China.
