# Supplementary figures and images for: Association of daytime napping with incidence of chronic kidney disease and end-stage kidney disease: A prospective observational study
Source: PLoS One. 2024 Mar 21;19(3):e0298375. doi: 10.1371/journal.pone.0298375 (PMC10956792; doi:10.1371/journal.pone.0298375)

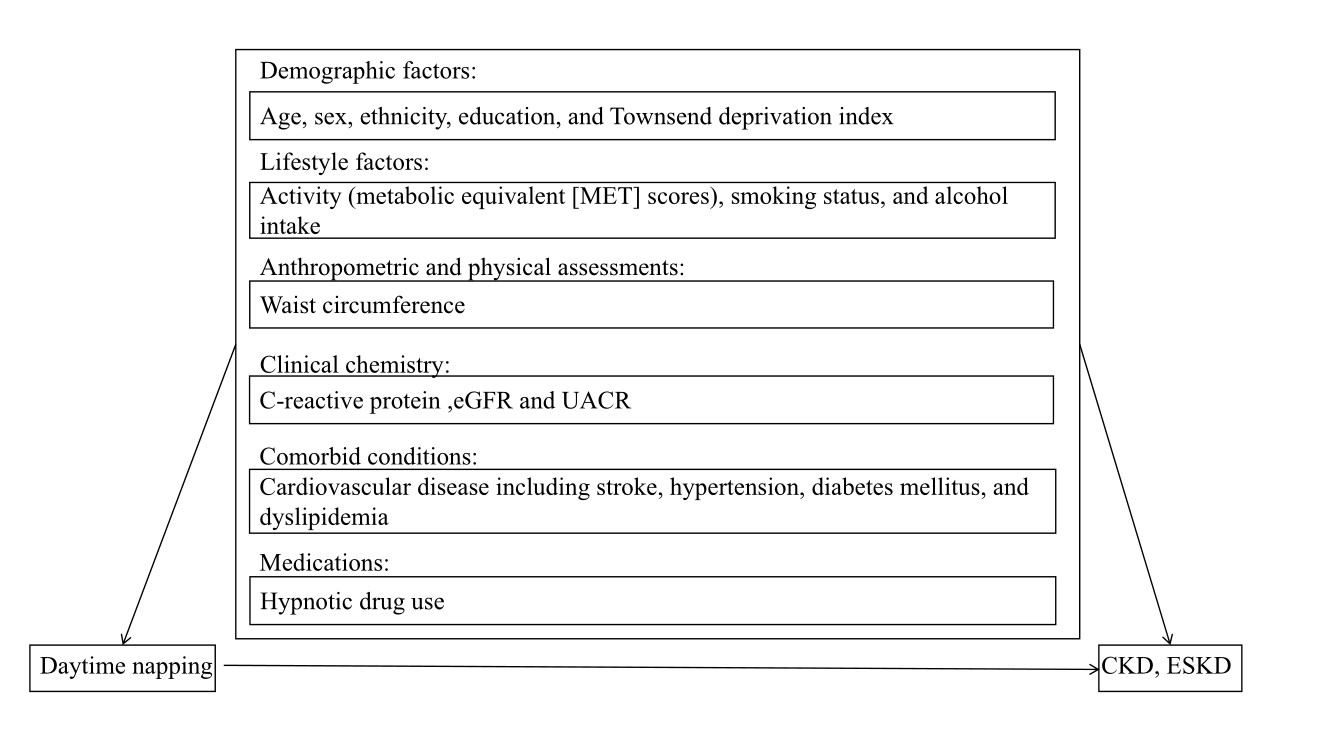

Supplement: S1 Fig — eGFR, estimated glomerular filtration rate; UACR, urinary protein creatinine ratio; CKD, chronic kidney disease; ESKD, end-stage kidney disease. (TIF) [file pone.0298375.s002.tif]
