# Supplementary material for: Association of daytime napping with incidence of chronic kidney disease and end-stage kidney disease: A prospective observational study
Source: PLoS One. 2024 Mar 21;19(3):e0298375. doi: 10.1371/journal.pone.0298375 (PMC10956792; doi:10.1371/journal.pone.0298375)
Supplement: S1 Table — (PDF) [file pone.0298375.s003.pdf]

S1 Table. ICD encoding of all variables in the UK Biobank.

**Variable**	**Data-Field**
ESRD	42026
Daytime napping	1190
HIV	130204-130213
Pregnancy	3140
Baseline age	21022
Sex	31
Race	21000
Education	6138
Townsend deprivation index (TDI)	189
Smoking status	20116
Alcohol consumption	20117
MET scores	22040
WC	48
Hypertension	131286
Diabetes mellitus	130706-130714<br>even number
Cardiovascular disease (CVD or stroke)	131270-131284;<br>131288-131378<br>even number
Dyslipidemia or Hyperlipidemia	30690(TC)<br>30760(HDL-C)<br>30780(LDL-C)<br>30870(TG)<br>41270(E78)
CRP	30710
eGFR	30720
UACR	30500
Clonazepam	1140872150
Lorazepam	1140863302
Alprazolam	1140863308
Dichloralphenazone	1140855824
Emepronium bromide	1140858082
Cetiprin	1140858084
Chloral hydrate product	1140863016
Heminevrin	1140863036
Rohypnol	1140863106
Flurazepam	1140863110
Dalmane	1140863112
Loprazolam	1140863120
Zopiclone	1140863144

Lormetazepam	1140863176
Nitrazepam	1140863182
Mogadon	1140863194
Temazepam	1140863202
Normison	1140863210
Zolpidem	1140865016
Clomethiazole	1140909798
Stilnoct	1140864916
Zimovane	1140928004
Zaleplon	1141171404
Sonata	1141171410
Amitriptyline	1140879616
Doxepin	1140867640
