# Supplementary material for: Association of daytime napping with incidence of chronic kidney disease and end-stage kidney disease: A prospective observational study
Source: PLoS One. 2024 Mar 21;19(3):e0298375. doi: 10.1371/journal.pone.0298375 (PMC10956792; doi:10.1371/journal.pone.0298375)
Supplement: S2 Table — Equation on the basis of cystatin C levels for estimating GFR. GFR, glomerular filtration rate. The CKD-EPI cystatin C equation (2012) can be expressed as a single equation: 133×min(Scys/0.8, 1)−0.499×max(Scys/0.8, 1)−1.328×0.996 Age [×0.932 if female], where Scys is serum cystatin C, min indicates the minimum of Scr/κ or 1, and max indicates the maximum of Scys/κ or 1. (PDF) [file pone.0298375.s004.pdf]

S2 Table. The Chronic Kidney Disease Epidemiology Collaboration (CKD-EPI) equation on the basis of cystatin C levels for estimating GFR.

Basis of Equation and Sex	Serum Cystatin C (mg/liter)	Equation for Estimating GFR
Female or Male	≤0.8	$133\times(\text{Scys}/0.8)^{-0.499}\times0.996^{\text{Age}}$ [×0.932 if female]
Female or Male	>0.8	$133\times(\text{Scys}/0.8)^{-1.328}\times0.996^{\text{Age}}$ [×0.932 if female]

GFR, glomerular filtration rate.

The CKD-EPI cystatin C equation (2012) can be expressed as a single equation: $133\times\min(\text{Scys}/0.8, 1)^{-0.499}\times\max(\text{Scys}/0.8, 1)^{-1.328}\times0.996^{\text{Age}}$ [×0.932 if female], where Scys is serum cystatin C, min indicates the minimum of Scr/κ or 1, and max indicates the maximum of Scys/κ or 1.
