# Supplementary material for: Association of daytime napping with incidence of chronic kidney disease and end-stage kidney disease: A prospective observational study
Source: PLoS One. 2024 Mar 21;19(3):e0298375. doi: 10.1371/journal.pone.0298375 (PMC10956792; doi:10.1371/journal.pone.0298375)
Supplement: S3 Table — TDI, Townsend deprivation index; MET, metabolic equivalent task; WC, waist circumference; CVD, cardiovascular disease; eGFR, estimated glomerular filtration rate; UACR, urinary protein creatinine ratio; CRP, C-Reactive Protein; CKD, chronic kidney disease; ESKD, end-stage kidney disease. level 1 = “uneducated”, level 2 = “O levels/GCSEs or equivalent” or “CSEs or equivalent”, level 3 = “A levels/AS levels or equivalent” or “NVQ or HND or HNC or equivalent” or “Other professional qualifications”, level 4 = “College or University degree”. Data are presented as mean + standard deviation or median (interquartile range) for continuous variables and number (percentage) for categorical variables. (PDF) [file pone.0298375.s005.pdf]

S3 Table. Baseline characteristics of the participants according to daytime napping with data after check in the UK Biobank.

	Daytime napping		
	Never/rarely (n=262,567)	Sometimes (n=177,268)	Usually (n=24,365)
Age (y)	56.0 [48.0, 62.0]	59.0 [51.0, 64.0]	61.00[54.0, 65.0]
Men(%)	108,526 (41.3)	88,770 (50.1)	15,964 (65.5)
Ethnicity			
Asian	5316 (2.0)	4743 (2.7)	765 (3.1)
Black	3570 (1.4)	3660 (2.1)	433 (1.8)
White	249,933 (95.2)	165,958 (93.6)	22,743 (93.3)
Others	3748 (1.4)	2907 (1.6)	424 (1.7)
Educational status			
Level1	36,919 (14.1)	35,764 (20.2)	6124 (25.1)
Level2	72,258 (27.5)	47,239 (26.6)	5918 (24.3)
Level3	60,271 (23.0)	41,533 (23.4)	5537 (22.7)
Level4	93,119 (35.5)	52,732 (29.7)	6786 (27.9)
TDI	-2.27 [-3.70, 0.24]	-1.97 [-3.55, 0.88]	-1.62 [-3.37, 1.53]
Smoking status			
Never	151,424 (57.7)	92,458 (52.2)	11,146 (45.7)
Previous	85,971 (32.7)	63,835 (36.0)	9605 (39.4)
Current	25,172 (9.6)	20,975 (11.8)	3614 (14.8)
Alcohol consumption			
Never	10,516 (4.0)	8518 (4.8)	1424 (5.8)
Previous	7738 (2.9)	7224 (4.1)	1582 (6.5)
Current	244,313 (93.0)	161,526 (91.1)	21,359 (87.7)
MET(m/w)	1786.0[824.0, 3546.0]	1760.0[787.5, 3576.0]	1680.0[688.5, 3591.0]
WC(cm)	88.0[79.0, 97.0]	92.0 [83.0, 101.0]	96.0 [87.0, 105.0]
Hypertension	58,505 (22.3)	54,205 (30.6)	9357 (38.4)

Diabetes Mellitus	8922 (3.4)	12,144 (6.9)	2679 (11.0)
CVD	22,380(8.5)	23,105 (13.0)	4872 (20.0)
Dyslipidemia	116,102 (44.2)	85,927 (48.5)	12,449 (51.1)
eGFR_scys	91.94 [80.08, 102.64]	87.18 [75.43, 99.24]	83.63 [71.89, 96.14]
UACR (mg/g)			
<30	252,420 (96.1)	167,891 (94.7)	22,648 (93.0)
30-300	9416 (3.6)	8480 (4.8)	1530 (6.3)
≥300	731 (0.3)	897 (0.5)	187 (0.8)
CRP (mg/L)	1.21 [0.60, 2.50]	1.47 [0.72, 3.03]	1.62 [0.78, 3.41]
Hypnotic drug use	5876 (2.2)	4870 (2.7)	1029 (4.2)
CKD event	12,582 (4.8)	13,193 (7.4)	2555 (10.5)
ESKD event	341 (0.1)	470 (0.3)	116 (0.5)

TDI, Townsend deprivation index; MET, metabolic equivalent task; WC, waist circumference; CVD, cardiovascular disease; eGFR, estimated glomerular filtration rate; UACR, urinary protein creatinine ratio; CRP, C-Reactive Protein; CKD, chronic kidney disease; ESKD, end-stage kidney disease. level 1 = "uneducated", level 2 = "O levels/GCSEs or equivalent" or "CSEs or equivalent", level 3 = "A levels/AS levels or equivalent" or "NVQ or HND or HNC or equivalent" or "Other professional qualifications", level 4 = "College or University degree".

Data are presented as mean ± standard deviation or median (interquartile range) for continuous variables and number (percentage) for categorical variables.
