# Supplementary material for: Association of daytime napping with incidence of chronic kidney disease and end-stage kidney disease: A prospective observational study
Source: PLoS One. 2024 Mar 21;19(3):e0298375. doi: 10.1371/journal.pone.0298375 (PMC10956792; doi:10.1371/journal.pone.0298375)
Supplement: S4 Table — CKD, chronic kidney disease; ESKD, end-stage kidney disease; CVD, cardiovascular disease; OSA, obstructive sleep apnea. Statistical analysis using Cox regression. Results are expressed as multivariable-adjusted hazard ratios and (95% confidence interval). All analyses are adjusted for basic age, sex, ethnicity, educational status, TDI, smoking status, alcohol consumption, MET scores, WC, hypnotic drug use, history of CVD, hypertension, diabetes mellitus and dyslipidemia, CRP, basic eGFR and UACR. (PDF) [file pone.0298375.s006.pdf]

S4 Table. Association between daytime napping and incident CKD or ESKD in participants without hypertension, diabetes mellitus, CVD, OSA and dyslipidemia at baseline.

	CKD		ESKD	
	**HR (95%CI)**	**P value**	**HR (95%CI)**	**P value**
Never	ref	ref	ref	ref
Sometimes	1.06 (1.00-1.14)	0.070	1.41 (0.95-2.08)	0.088
Usually	1.06 (0.91-1.23)	0.456	0.99 (0.39-2.52)	0.984
P for trend	1.04 (0.94-1.16)	0.456	0.99 (0.51-1.92)	0.984

CKD, chronic kidney disease; ESKD, end-stage kidney disease; CVD, cardiovascular disease; OSA, obstructive sleep apnea.

Statistical analysis using Cox regression. Results are expressed as multivariable-adjusted hazard ratios and (95% confidence interval).

All analyses are adjusted for basic age, sex, ethnicity, educational status, TDI, smoking status, alcohol consumption, MET scores, WC, hypnotic drug use, history of CVD, hypertension, diabetes mellitus and dyslipidemia, CRP, basic eGFR and UACR.
