# Supplementary material for: Association of daytime napping with incidence of chronic kidney disease and end-stage kidney disease: A prospective observational study
Source: PLoS One. 2024 Mar 21;19(3):e0298375. doi: 10.1371/journal.pone.0298375 (PMC10956792; doi:10.1371/journal.pone.0298375)
Supplement: S6 Table — Statistical analysis using Cox regression. Results are expressed as multivariable-adjusted hazard ratios and (95% confidence interval). All analyses are adjusted for basic age, sex, ethnicity, educational status, TDI, smoking status, alcohol consumption, MET scores, WC, hypnotic drug use, history of CVD, hypertension, diabetes mellitus and dyslipidemia, CRP, basic eGFR and UACR. (PDF) [file pone.0298375.s008.pdf]

S6 Table. Association between daytime napping and incident CKD or ESKD in competing-risk of death models.

	CKD		ESKD	
	**HR (95%CI)**	**P value**	**HR (95%CI)**	**P value**
Never	ref	ref	ref	ref
Sometimes	1.07 (1.04-1.11)	<0.0001	1.08 (0.93-1.25)	0.311
Usually	1.08 (1.02-1.15)	0.014	1.19(0.96-1.49)	0.112
P for trend	1.06 (1.01-1.10)	0.014	1.13(0.97-1.32)	0.112

Statistical analysis using Cox regression. Results are expressed as multivariable-adjusted hazard ratios and (95% confidence interval).

All analyses are adjusted for basic age, sex, ethnicity, educational status, TDI, smoking status, alcohol consumption, MET scores, WC, hypnotic drug use, history of CVD, hypertension, diabetes mellitus and dyslipidemia, CRP, basic eGFR and UACR.
