# Supplementary material for: Association of daytime napping with incidence of chronic kidney disease and end-stage kidney disease: A prospective observational study
Source: PLoS One. 2024 Mar 21;19(3):e0298375. doi: 10.1371/journal.pone.0298375 (PMC10956792; doi:10.1371/journal.pone.0298375)
Supplement: Attachment — Submitted filename: Response to Reviewers.docx [file pone.0298375.s010.docx]

**Rebuttal Letter**

Dec 23, 2023

Dear Editor,

We sincerely thank your insightful feedback and suggestions on our manuscript titled “Association of daytime napping with incidence of chronic kidney disease and end-stage kidney disease: a prospective observational study”. We greatly appreciate this chance to further refine and enhance our work.

In response, we have diligently addressed each comment and made the necessary revisions. All changes have been highlighted within the manuscript. Please do not hesitate to point out any further issues or concerns.

Best regards,

Guoyuan Lu

Journal Requirements:

Please review your reference list to ensure that it is complete and correct. If you have cited papers that have been retracted, please include the rationale for doing so in the manuscript text, or remove these references and replace them with relevant current references. Any changes to the reference list should be mentioned in the rebuttal letter that accompanies your revised manuscript. If you need to cite a retracted article, indicate the article’s retracted status in the References list and also include a citation and full reference for the retraction notice.

R：Thank you for your reminder. we have already reviewed the reference list to ensure that it is complete and correct. At the same time, a citation has been added to the discussion section of the article, resulting in a modification to the reference list, as shown below:

“In contrast, a study from iranian ravansar cohort demonstrated that after adjusting for confounding factors, no statistically significant association of daytime napping with CKD was observed among Iranian adults in the Kurdish region [46].” **(P24)**

“46. Hemati N, Shiri F, Ahmadi F, Najafi F, Moradinazar M, Norouzi E, Khazaie H.Association between sleep parameters and chronic kidney disease: findings

from iranian ravansar cohort study. BMC Nephrol. 2023 May 17;24(1):136.

doi: 10.1186/s12882-023-03177-3.” **(P34)**

Reviewer #2: The reviewer gladly realized that the suggestions have been accepted and the manuscript has been accordingly modified and improved.
The reviewer finally suggests to take care of these few points before final acceptance.

Final suggestions:
1) At page 10, in the final line the colon is repeated.

R: Thank you for pointing that out. We have already removed the unnecessary colon at the end of the last line on page 11.

2) At page 17, the sentence “In the full model, with further adjustments, although the estimates were showed a gradual attenuation, the association between napping an elevated risk of CKD remained consistent” is better rephrased: “In the full model, with further adjustments, although the estimates showed a meaningful attenuation, the association between napping an elevated risk of CKD remained consistent”
(please note that the decrease from 1.90 to 1.09 is pretty large!)

R: Thank you for your comprehensive examination of our manuscript. We have made the following modifications based on your suggestions：

“In the full model, with further adjustments, although the estimates showed a meaningful attenuation, the association between napping and elevated risk of CKD remained consistent (P for trend = .004). ” **(P17)**

3) At page 17, the sentence “In the full model, there was no consistent association found between napping and an elevated risk of ESKD (P for trend =.060).” is better rephrased, for example: “In the full model, association was found between napping and elevated risk of ESKD as well. However, the P-values (0.377 and 0.060, respectively) do not allow to exclude the effect of confounders embedded in the data. Therefore, an association between napping and elevated risk of ESKD cannot be asserted”

R: Thank you for your thorough review of our manuscript. Your expression is very reasonable. We have already made modifications in accordance with your suggestions, as follows:

“In the full model, after adjusting for covariates in model 3, relative to the reference group, the HRs (95% CIs) for ESKD were 1.07 (0.92 to 1.24) in the group that occasionally naps and 1.24 (0.99 to 1.54) in the group that usually naps (as illustrated in Fig 2). A correlation was observed between napping and an increased risk of ESKD as well. Nevertheless, the P-values (0.377 and 0.060, respectively) did not allow to exclude the effect of confounding factors embedded in the data. Therefore, an association between napping and elevated risk of ESKD could not be asserted.” **(P17-18)**

“After adjusting for these factors, no statistically significant association of daytime napping with ESKD was observed, indicating that these factors might confound the observed associations between daytime napping and ESKD.” **(P27)**

4) At page 23, during the discussion session, it would be beneficial to add a few lines where the authors highlight their findings of an association between napping and both CKD and ESKD and that the P-values where applied as element of acceptance or rejection of the association.

R: Thank you for your feedback. We appreciate your attention to detail. We have revised the content in the discussion section as per your suggestions, as follows:

“Our study revealed a positive association between daytime napping and an elevated risk of incident CKD. After adjusting for confounding factors, although their relationship weakened, the p-value for trend still hold statistical significance. The variations in these findings might stem from differences among participants in terms of race, lifestyle, and health. Consequently, future studies should take into account the varying risk levels for CKD across diverse racial and ethnic groups.” **(P24)**

“After adjusting for confounding factors, the P-value for trend (= 0.060) was no longer statistically significant. Therefore, it could not be conclusively stated that a relationship exists between napping and an increased risk of ESKD.” **(P26)**

5) At page 28, the statement “These findings suggested that napping was an unrecognized and clinically significant risk factor for incident CKD” is a strong conclusion that cannot be supported by the presented data. The reviewer recommends its removal.

R: Thank you for the excellent suggestion. We appreciate your perspective and agree with your point. We have made the necessary revisions in the conclusion section to reflect this, as follows:

“In conclusion, self-reported daytime napping was positively associated with an increased incident CKD risk, suggesting that napping may be a potential risk factor for the onset of CKD. However, the specific mechanism by which daytime napping induces the occurrence of CKD is not well understood; Future work is needed to explore the specific mechanisms and assess whether the observed relationship between napping with incident CKD is causal.” **(P28)**

Reviewer #3:

I trust this message finds you well. I have reviewed your manuscript titled "**Association of daytime napping with incidence of chronic kidney disease and end-stage kidney disease: a prospective observational study**" and found it to be intriguing and well-articulated.

R：Thank you very much for your affirmation and recognition of the article titled “Association of daytime napping with incidence of chronic kidney disease and end-stage kidney disease: a prospective observational study.” Your affirmation and recognition have further boosted our confidence. We will implement further edits to the article as per your suggestions to enhance its overall clarity and expression.

In the context of your research on the association between daytime napping and chronic kidney disease (CKD), I would like to draw your attention to a recent study that might complement and strengthen your findings. The study is titled "**Association between sleep parameters and chronic kidney disease: findings from Iranian Ravansar Cohort Study**" by Hemati et al. published in BMC Nephrology (DOI: 10.1186/s12882-023-03177-3).

The Iranian Ravansar Cohort Study provides valuable insights into the relationship between sleep parameters and CKD, offering potential additional evidence to support your investigation. Considering the alignment in the research focus, incorporating findings from this Iranian study could enhance the comprehensiveness of your analysis and contribute to a more robust interpretation of the association between daytime napping and CKD.

I recommend reviewing the mentioned study and evaluating the possibility of integrating relevant insights into your work.

R：Thank you for providing us with detailed guidance and advice. We have carefully read and understood the content of the article titled "**Association between sleep parameters and chronic kidney disease: findings from Iranian Ravansar Cohort Study**" based on your suggestions. Accordingly, we have made relevant modifications in line with the results and discussions of the article. The revisions provide a stronger explanation of the relationship between daytime napping and CKD. The modified content is as follows:

“In contrast, a study from iranian ravansar cohort demonstrated that after adjusting for confounding factors, no statistically significant association of daytime with CKD was observed among Iranian adults in the Kurdish region [46]. Even after accounting for potential confounding factors, our study still indicated an association between daytime napping and an increased risk of developing CKD. Despite a weakened association, the p-value for the trend (= .040) remains statistically significant. The variations in these findings might stem from differences among participants in terms of race, lifestyle, and health. Consequently, future studies should take into account the varying risk levels for CKD across diverse racial and ethnic groups.” **(P24)**
